# Increased risk of incident dementia associated with vitamin D deficiency in glaucoma patients: a TriNetX cohort study

**DOI:** 10.3389/fnut.2026.1760959

**Published:** 2026-02-10

**Authors:** Yu-Chen Cheng, Chien-Lin Lu, Joshua Wang, Ming Ling Tsai, Kuo-Cheng Lu

**Affiliations:** 1 School of Medicine, College of Medicine, Fu Jen Catholic University, New Taipei City, Taiwan; 2 Department of Neurology, Fu Jen Catholic University Hospital, Fu Jen Catholic University, New Taipei City, Taiwan; 3 Division of Nephrology, Department of Internal Medicine, Fu Jen Catholic University Hospital, Fu Jen Catholic University, New Taipei City, Taiwan; 4 Department of Research, Taipei Tzu Chi Hospital, Buddhist Tzu Chi Medical Foundation, New Taipei City, Taiwan; 5 School of Biomedical Sciences, Queensland University of Technology, Brisbane, QLD, Australia; 6 Department of Ophthalmology, Taipei Buddhist Tzu Chi Hospital, Buddhist Tzu Chi Medical Foundation, New Taipei City, Taiwan; 7 Division of Nephrology, Department of Medicine, Taipei Tzu Chi Hospital, Buddhist Tzu Chi Medical Foundation, New Taipei City, Taiwan

**Keywords:** cognitive decline, dementia, glaucoma, neurodegeneration, vitamin D deficiency

## Abstract

**Introduction:**

Glaucoma is a progressive optic neuropathy associated with increased neurodegenerative risk. Vitamin D Deficiency (VDD) is a widespread systemic factor linked to neurobiological dysfunction. This study investigated the longitudinal association between VDD and the 5-year incidence of neurodegenerative outcomes in this glaucoma population.

**Methods:**

This retrospective cohort study used a large electronic health records (EHRs) network. Glaucoma patients were classified as VDD (< 30 ng/mL) or vitamin D adequate (VDA) (≥30 ng/mL). A 1:1 propensity score matching (PSM) procedure matched 10,881 patients per cohort based on 47 covariates. The primary endpoints were the 5-year incidence of unspecified dementia, Alzheimer disease (AD), and Parkinson disease (PD), analyzed using Cox proportional hazards models.

**Results:**

After PSM, VDD was associated with a higher 5-year risk of unspecified dementia (HR 1.241, 95% CI 1.066–1.446; *p* = 0.005), with greater risk in severe deficiency (< 20 ng/mL) (HR 1.493, 95% CI 1.179–1.890; *p* < 0.001). No association was found between VDD and AD or PD. Major predictors included advanced age (HR 6.84), hypertension (HR 2.70), hypoalbuminemia (HR 2.64), elevated CRP (HR 1.38), and diabetes (HR 1.29). Removing long-term NSAID users reduced the dementia risk to non-significant levels (HR 1.178, 95% CI: 0.998–1.391, *p* = 0.053), suggesting NSAID anti-inflammatory effects may not counteract VDD-related dementia risk in glaucoma patients.

**Conclusion:**

VDD is significantly associated with an increased risk of developing dementia in patients with glaucoma. These time-dependent and dose-response findings raise the possibility that correcting vitamin D deficiency may influence neurodegenerative outcomes, additional prospective studies are required to establish causality and clarify clinical implications.

## 1 Introduction

The global rise in age-related neurological conditions establishes neurodegeneration as a major public health priority (1). Glaucoma, the world's leading cause of irreversible blindness, presents a significant and increasing clinical burden. In 2020, the number of people aged 40–80 years affected by glaucoma globally was estimated at 76 million, projected to rise to 111.8 million by 2040 (2, 3). Glaucoma, historically defined by abnormal intraocular pressure, is now fundamentally recognized as a progressive optic neuropathy involving the irreversible attrition of retinal ganglion cells (RGCs) and their axons (4, 5). This pathology is strongly supported by shared molecular mechanisms with central nervous system (CNS) diseases, including oxidative stress, chronic neuroinflammation, mitochondrial damage, and common genetic risk loci (5–8). This mechanistic overlap suggests that glaucoma should not be viewed as an isolated ocular disorder but as part of a broader, systemic neurodegenerative vulnerability (9).

This systemic view is further validated by population-based epidemiological data. Glaucoma patients consistently demonstrate a heightened risk for developing general cognitive impairment and Alzheimer's disease (AD) compared to the general population, with established hazard ratios (HRs) typically ranging from 1.23 to 1.89 (10–13). This association emphasizes the fact that glaucoma patients represent a cohort already burdened with a pre-existing neurodegenerative process, making them uniquely susceptible to additional systemic factors that influence central neurodegenerative trajectories (9). However, the exact role of systemic factors in influencing the longitudinal hazard of developing central neurodegenerative diseases in this high-risk population has not been fully elucidated. Establishing such associations is critical for treating glaucoma as a multisystem disorder and for informing preventative strategies and interdisciplinary management in this aging population.

One such crucial and widespread systemic factor influencing these shared trajectories is vitamin D Deficiency (VDD), defined in this study as serum 25-hydroxyvitamin D (25(OH)D) levels < 30 ng/mL. Vitamin D's physiological influence extends far beyond its well-known role in calcium homeostasis (14). The active metabolite, 1,25-dihydroxyvitamin D, functions as a pleiotropic steroid hormone, binding the vitamin D Receptor (VDR), which is widely expressed in key CNS areas, including the retina, optic nerve head, and brain parenchyma (15–17). VDD has been implicated in diverse neurobiological processes, such as regulating immune responses (18, 19), stabilizing vascular endothelial function (20), and promoting the clearance of amyloid-beta (21–23). Vitamin D deficiency is linked to a higher risk of dementia and Alzheimer's disease, with low levels increasing dementia risk by about 49% (24). Accordingly, a substantial body of observational evidence independently links VDD to an elevated risk of all-cause dementia in the general population, with meta-analyses reporting HRs between 1.19 and 2.28 (25–27).

Although the neurodegenerative risks conferred by both glaucoma and VDD are individually well-characterized, the synergistic impact of VDD specifically on the longitudinal neurodegenerative risk within a cohort already compromised by glaucoma remains a significant clinical knowledge gap. Glaucoma patients offer a critical model to evaluate if a modifiable systemic factor like VDD accelerates pre-existing CNS decline.

Therefore, we conducted a large-scale, retrospective cohort study utilizing Propensity Score Matching (PSM) to assess the longitudinal association between baseline VDD and the subsequent 5-year incidence of three key neurodegenerative outcomes: unspecified dementia, AD, and Parkinson's disease (PD), in patients with glaucoma. By employing comprehensive PSM to create two highly comparable VDD and vitamin D Adequacy (VDA, ≥30 ng/mL) cohorts, we aimed to minimize the effects of systemic confounding. We hypothesized that VDD would function as a potential risk marker associated with a significantly higher hazard of neurodegenerative disease, particularly dementia, in this high-risk patient group.

## 2 Methods

### 2.1 Study design and data source

This investigation was conducted as a retrospective cohort study, utilizing de-identified, and patient-level data obtained from the TriNetX platform. TriNetX is a federated global health research network that aggregates real-world electronic health records (EHRs) from hospitals and healthcare systems across the United States. The analysis was executed within the US Collaborative Network on October 11, 2025, which encompasses 71 healthcare organizations providing comprehensive longitudinal clinical data. All data extraction and analyses were performed exclusively within the secure TriNetX cloud environment. Given that all data were fully de-identified in strict compliance with HIPAA and GDPR, the requirement for institutional review board approval and informed patient consent was waived by the platform. Nevertheless, the study protocol received independent review and approval from the Taipei Tzu Chi Hospital Institutional Review Board (Approval Number: 14-IRB134) and adhered to the ethical principles of the Declaration of Helsinki.

### 2.2 Study population and exposure definition

The initial population comprised adults (aged 18 years or older) with a documented glaucoma diagnosis (ICD-10-CM codes H40–H42, specifically including primary open-angle H40.1 and primary angle-closure H40.2) recorded between 1 January 2005 and 1 January 2020. The initial screen identified 761,232 patients. Cases were subsequently restricted to first-occurrence glaucoma without secondary causes by excluding any secondary glaucoma codes (H40.3–H40.6) recorded within 1 year on or before the index glaucoma diagnosis, resulting in 722,862 eligible patients.

Vitamin D status was determined based on serum 25-hydroxyvitamin D [25(OH)D] concentrations identified by LOINC code 1989-3. Measurements obtained within 1 year prior to the first glaucoma record were included, and for patients with multiple results, the value closest to the index date was selected to best reflect pre-observation physiological status. To ensure stable baseline exposure, individuals with any historical 25(OH)D value < 30 ng/mL were excluded from the VDA cohort, and those with any prior value ≥30 ng/mL were excluded from the VDD cohort, minimizing potential misclassification due to recent status changes. Over 92% of matched participants in both cohorts had complete baseline 25(OH)D data with minimal missing values. Patients were categorized into two exposure groups: VDD (< 30 ng/mL) and VDA (≥30 ng/mL), with 16,602 and 15,638 participants, respectively, prior to matching.

### 2.3 Index date and follow-up period

The index date was set as the earliest date a patient met all the criteria for a glaucoma diagnosis. The observational follow-up period commenced 1 day after the index date, with a maximum duration of 1,825 days (5 years), censored by the event of death or the end of available health records, whichever occurred first. Any patient with evidence of the primary outcome prior to the start of the observation window was excluded from the analyses to ensure the capture of incident outcomes.

### 2.4 Propensity score matching

To mitigate selection bias and minimize the influence of confounding factors, a 1:1 PSM procedure was implemented using a greedy nearest-neighbor algorithm with a caliper width of 0.1. Matching covariates included 46 clinically relevant variables, such as demographic features, comorbid cardiometabolic conditions, medication use, renal and metabolic laboratory indices, and inflammatory biomarkers. Crucially, the exposure-defining variable (serum 25(OH)D levels) was excluded from the covariate list to maintain the primary exposure contrast. First, the standardized mean differences (SMDs) were calculated post-matching to confirm acceptable covariate balance, with an SMD < 0.1 considered indicative of comparability. Second, the overlap of propensity score distributions (common support) was visually inspected to ensure that the matched cohort shared similar characteristics across the entire range of the propensity scores (Supplementary Figure S1). The final pre-matched cohorts comprised 16,602 patients in VDD and 15,638 in VDA. Following PSM, both the VDD and VDA cohorts contained 10,881 individuals.

### 2.5 Outcome measures

The primary endpoint was the incidence of neurodegenerative disorders during the 5-year follow-up period, defined as a composite outcome comprising the following ICD-10-CM codes: unspecified dementia (F03), Alzheimer's disease (G30), and Parkinson's disease (G20). Unspecified dementia captured clinically confirmed dementia without subtype specification, Alzheimer's disease included all sub codes representing Alzheimer's pathology—the most common specific dementia subtype—and Parkinson's disease represented primary Parkinsonism. These conditions were selected for their clinical relevance and high prevalence in U.S.-based EHR data, with Alzheimer's disease and unspecified dementia together accounting for most dementia diagnoses and providing a robust basis for evaluating associations with vitamin D status.

### 2.6 Statistical and sensitivity analysis

All statistical computations, including the estimation of absolute risks, risk ratios, and odds ratios, were executed using the integrated Risk Analysis features of the TriNetX platform. The cumulative incidence of time-to-event outcomes was visualized using Kaplan–Meier survival curves, and differences between the VDD and VDA groups were tested using the Log-Rank test. Cox proportional hazards models were subsequently employed to calculate the HRs and their associated 95% Confidence Intervals (CIs) for incident outcomes. Statistical significance was predefined by a two-sided *p*-value less than 0.05. Primary statistical analyses, including propensity score matching (PSM) and Cox proportional hazards regression, were performed within the TriNetX (Cambridge, MA, USA) analytics platform. For data visualization and figure generation, we utilized GraphPad Prism (version 8.0.1) and Microsoft Excel.

A comprehensive set of sensitivity analyses and one exploratory analysis were performed. We first conducted a Landmark Analysis to assess the time-varying effect of VDD on cumulative incidence and HRs at 1, 3, and 5 years post-index. For Confounding Assessment, we compared the unadjusted HRs with the primary PSM-matched HRs, which quantified the impact of controlling for confounders. We then performed a Severe Deficiency Adjustment by re-analyzing the data using a stricter classification for VDD, defined as 25(OH)D levels < 20 ng/mL, to investigate a potential dose-response relationship. To evaluate the potential mitigating effect of systemic anti-inflammation, we performed a non-steroidal anti-Inflammatory drugs (NSAID) confounding assessment by excluding individuals with recorded long-term NSAID use. Finally, an exploratory healthcare utilization analysis was performed by comparing the mean number of hospital visits between the matched VDD and VDA cohorts to assess baseline differences in health-seeking behavior or comorbidity burden.

To evaluate the potential influence of detection bias and systemic health disparities on study outcomes, we performed supplementary analyses of healthcare utilization over the 5-year follow-up period. Specifically, we compared the VDD and VDA cohorts in terms of hospital visit counts (Supplementary Table S5), frequency of all-cause hospitalizations (Supplementary Table S6), and utilization of ophthalmology-specific services and procedures (Supplementary Table S7), using independent *t*-tests and Chi-square tests. Finally, to address potential selection bias from missing data, we conducted a Restricted PSM sensitivity analysis by limiting matching covariates to 26 high-completeness variables (>85% data availability), ensuring that the observed association was not sensitive to less complete laboratory parameters (Supplementary Table S8).

## 3 Results

### 3.1 Cohort characteristics and propensity score matching

**Figure 1:**
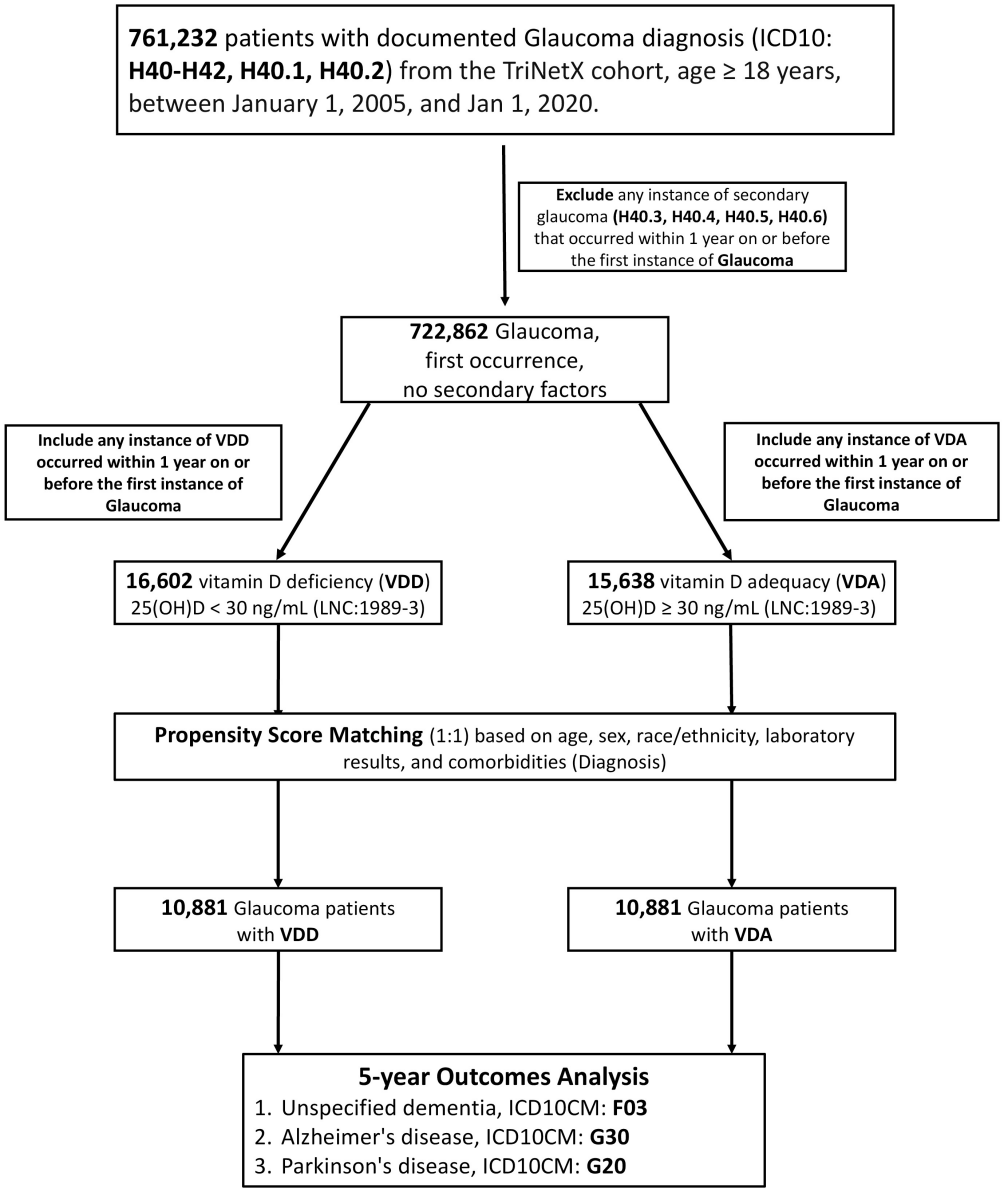
Study flow diagram: cohort assembly and propensity-score matching (PSM) of glaucoma patients by vitamin D status. This schematic illustrates the analytic process used to construct the study cohorts from a retrospective dataset within the TriNetX research network, designed to evaluate the 5-year incidence risks of neurodegenerative diseases. The process began with an initial source population of 761,232 adults (aged 18 years or older) diagnosed with glaucoma (ICD-10 codes: H40–H42, specifically H40.1 and H40.2) recorded between 1 January 2005 and 1 January 2020. This group was subsequently refined to 722,862 individuals by excluding those with any secondary causes of glaucoma (H40.3–H40.6) recorded within the year preceding or on the index diagnosis date. Exposure groups were defined based on serum 25-hydroxyvitamin D (25(OH)D) levels (LOINC 1989-3) measured up to 1 year before the initial glaucoma diagnosis. This classification established two principal groups: the Vitamin D Deficiency (VDD) cohort (25(OH)D less than 30 ng/mL, *n* = 16,602) and the Vitamin D Adequacy (VDA) cohort (30 ng/mL or greater, *n* = 15,638). To ensure baseline comparability, a 1:1 Propensity Score Matching (PSM) procedure was employed. This matching utilized covariate including demographics (age, sex, race/ethnicity), relevant laboratory findings, and comorbid conditions. The final, balanced study cohorts used for comparative analysis consisted of 10,881 patients in the VDD group and 10,881 patients in the VDA group. The pre-specified 5-year outcomes analyzed were incident unspecified Dementia (ICD-10 F03), Alzheimer's disease (G30), and Parkinson's disease (G20).

As shown in Figure 1, the derivation of the final study cohort commenced with the identification of 761,232 adult patients (aged 18 years or older) diagnosed with glaucoma (ICD-10 codes H40–H42). After applying exclusion criteria for secondary glaucoma, 722,862 individuals remained. Vitamin D status was determined by serum 25-hydroxyvitamin D (25(OH)D) levels: 16,602 individuals met the criteria for VDD (25(OH)D < 30 ng/mL), and 15,638 for VDA (25(OH)D ≥ 30 ng/mL).

To ensure unbiased comparisons, a comprehensive 1:1 PSM was performed using 46 covariates (excluding the exposure-defining variable, serum 25(OH)D), successfully creating two well-balanced cohorts, each containing 10,881 patients. As shown in Table 1, the matching process successfully resolved marked baseline imbalances, including the age difference (VDD 61.5 ± 11.3 years vs. VDA 61.6 ± 11.5 years; Standardized Mean Difference, SMD, SMD = 0.002). Furthermore, the propensity score density plots demonstrated near-perfect overlap between the matched cohorts, confirming robust common support (Supplementary Figure S1). These finalized cohorts were then followed for up to 5 years to assess the association of neurodegenerative outcomes.

**Table 1:** Glaucoma patient characteristics by vitamin D status, before and after PSM.

**Characteristics**	**Before matching**				**After matching**			
	**Means**±**SD**	**Patient count**	**% of Cohort**	**Std. diff**.	**Means**±**SD**	**Patient count**	**% of cohort**	**Std. diff**.
**Demographics**								
Age at index	57.819 ± 12.783 vs. 64.081 ± 11.140	16,602 vs. 15,638	100.00% vs. 100.00%	0.522	61.542 ± 11.335 vs. 61.565 ± 11.507	10,881 vs. 10,881	100.00% vs. 100.00%	0.002
Male		5,585 vs. 4,424	33.64% vs. 28.29%	0.116		3,325 vs. 3,337	30.56% vs. 30.67%	0.002
Female		11,016 vs. 11,212	66.35% vs. 71.70%	0.116		7,555 vs. 7,544	69.43% vs. 69.33%	0.002
White		8,227 vs. 10,379	49.55% vs. 66.37%	0.346		6,393 vs. 6,444	58.75% vs. 59.22%	0.010
Black or African American		4,932 vs. 2,318	29.71% vs. 14.82%	0.364		2,233 vs. 2,205	20.52% vs. 20.27%	0.006
Hispanic or Latino		2,705 vs. 1,160	16.29% vs. 7.42%	0.277		1,103 vs. 1,094	10.14% vs. 10.05%	0.003
Not Hispanic or Latino		13,041 vs. 13,721	78.55% vs. 87.74%	0.247		9,241 vs. 9,231	84.93% vs. 84.84%	0.003
Unknown Race		1,538 vs. 1,266	9.26% vs. 8.10%	0.042		1,018 vs. 1,018	9.36% vs. 9.36%	0.000
Asian		917 vs. 1,060	5.52% vs. 6.78%	0.052		718 vs. 709	6.60% vs. 6.52%	0.003
**Diagnosis**								
Hypertensive diseases		9,028 vs. 8,103	54.38% vs. 51.82%	0.051		5,640 vs. 5,684	51.83% vs. 52.24%	0.008
Diabetes mellitus		6,006 vs. 4,052	36.18% vs. 25.91%	0.223		3,207 vs. 3,255	29.47% vs. 29.91%	0.010
Ischemic heart diseases		1,841 vs. 1,579	11.09% vs. 10.10%	0.032		1,100 vs. 1,141	10.11% vs. 10.49%	0.012
Cerebrovascular diseases		999 vs. 946	6.02% vs. 6.05%	0.001		655 vs. 653	6.02% vs. 6.00%	0.001
**Medications**								
Antilipemic Agents		6,009 vs. 5,851	36.19% vs. 37.41%	0.025		3,917 vs. 3,907	36.00% vs. 35.91%	0.002
Diuretics		4,933 vs. 3,917	29.71% vs. 25.05%	0.105		2,915 vs. 2,971	26.79% vs. 27.30%	0.012
Blood Glucose Regulation Agents		5,486 vs. 3,650	33.04% vs. 23.34%	0.217		2,891 vs. 2,934	26.57% vs. 26.96%	0.009
Beta Blockers/Related		4,324 vs. 3,821	26.05% vs. 24.43%	0.037		2,680 vs. 2,744	24.63% vs. 25.22%	0.014
Ace Inhibitors		3,862 vs. 2,822	23.26% vs. 18.05%	0.129		2,133 vs. 2,196	19.60% vs. 20.18%	0.015
Calcium Channel Blockers		3,414 vs. 2,823	20.56% vs. 18.05%	0.064		2,024 vs. 2,038	18.60% vs. 18.73%	0.003
Angiotensin II Inhibitor		2,093 vs. 2,122	12.61% vs. 13.57%	0.029		1,404 vs. 1,399	12.90% vs. 12.86%	0.001
**Laboratory results**								
Calcidiol ng/mL	19.490 ± 6.597 vs. 42.917 ± 12.584	15,558 vs. 14,734	93.71% vs. 94.22%	2.332	20.450 ± 6.400 vs. 42.408 ± 12.556	10,076 vs. 10,253	92.60% vs. 94.23%	2.203
Potassium	4.202 ± 0.440 vs. 4.244 ± 0.427	14,857 vs. 13,909	89.49% vs. 88.94%	0.097	4.216 ± 0.429 vs. 4.228 ± 0.433	9,585 vs. 9,615	88.09% vs. 88.36%	0.029
Urea nitrogen	17.378 ± 11.533 vs. 17.837 ± 9.393	14,848 vs. 13,894	89.44% vs. 88.85%	0.044	17.193 ± 9.995 vs. 17.705 ± 9.716	9,573 vs. 9,610	87.98% vs. 88.32%	0.052
Calcium	9.350 ± 0.556 vs. 9.478 ± 0.502	14,822 vs. 13,964	89.28% vs. 89.30%	0.243	9.407 ± 0.531 vs. 9.447 ± 0.513	9,572 vs. 9,644	87.97% vs. 88.63%	0.078
0–8.5 mg/dL		2,228 vs. 1,207	13.42% vs. 7.72%	0.186		990 vs. 1,041	9.10% vs. 9.57%	0.016
8.5–10 mg/dL		13,701 vs. 12,688	82.53% vs. 81.14%	0.036		8,802 vs. 8,844	80.89% vs. 81.28%	0.010
10–11 mg/dL		2,720 vs. 3,358	16.38% vs. 21.47%	0.130		2,012 vs. 2,068	18.49% vs. 19.01%	0.013
11–13 mg/dL		232 vs. 205	1.40% vs. 1.31%	0.007		144 vs. 153	1.32% vs. 1.41%	0.007
Sodium	139.204 ± 2.825 vs. 139.545 ± 2.880	14,830 vs. 13,876	89.33% vs. 88.73%	0.119	139.429 ± 2.771 vs. 139.526 ± 2.827	9,566 vs. 9,595	87.92% vs. 88.18%	0.035
Bicarbonate	26.478 ± 3.106 vs. 26.876 ± 2.883	14,771 vs. 13,821	88.97% vs. 88.38%	0.133	26.633 ± 3.011 vs. 26.781 ± 2.924	9,524 vs. 9,561	87.53% vs. 87.87%	0.050
Bicarbonate		14,771 vs. 13,821	88.97% vs. 88.38%	0.019		9,524 vs. 9,561	87.53% vs. 87.87%	0.010
Creatinine	1.116 ± 1.908 vs. 1.065 ± 2.296	14,660 vs. 13,872	88.30% vs. 88.71%	0.024	1.038 ± 1.693 vs. 1.071 ± 1.791	9,514 vs. 9,544	87.44% vs. 87.71%	0.019
Glucose	121.12 ± 58.84 vs. 108.81 ± 39.60	14,771 vs. 13,794	88.97% vs. 88.21%	0.245	113.253 ± 47.13 vs. 111.650 ± 43.65	9,504 vs. 9,546	87.34% vs. 87.73%	0.035
0–60 mg/dL		620 vs. 345	3.73% vs. 2.21%	0.090		278 vs. 296	2.56% vs. 2.72%	0.010
60–90 mg/dL		6,292 vs. 5,793	37.90% vs. 37.04%	0.018		4,027 vs. 4,039	37.01% vs. 37.12%	0.002
90–120 mg/dL		9,646 vs. 9,707	58.10% vs. 62.07%	0.081		6,428 vs. 6,479	59.08% vs. 59.54%	0.010
120–150 mg/dL		4,929 vs. 3,697	29.69% vs. 23.64%	0.137		2,796 vs. 2,854	25.70% vs. 26.23%	0.012
150–180 mg/dL		3,403 vs. 2,194	20.50% vs. 14.03%	0.172		1,780 vs. 1,774	16.36% vs. 16.30%	0.001
Alanine aminotransferase	26.616 ± 38.096 vs. 24.809 ± 28.848	13,547 vs. 12,804	81.60% vs. 81.88%	0.053	24.574 ± 18.063 vs. 25.595 ± 33.536	8,761 vs. 8,779	80.52% vs. 80.68%	0.038
Hematocrit	39.590 ± 5.172 vs. 40.265 ± 4.554	13,632 vs. 12,636	82.11% vs. 80.80%	0.139	39.915 ± 4.852 vs. 40.113 ± 4.711	8,721 vs. 8,747	80.15% vs. 80.39%	0.041
Aspartate aminotransferase	26.255 ± 119.55 vs. 25.257 ± 30.02	13,448 vs. 12,682	81.00% vs. 81.10%	0.011	24.270 ± 13.49 vs. 25.553 ± 35.35	8,672 vs. 8,712	79.70% vs. 80.07%	0.048
Platelets	245.247 ± 77.24 vs. 239.359 ± 69.38	13,510 vs. 12,501	81.38% vs. 79.94%	0.080	242.020 ± 74.17 vs. 240.46 ± 70.64	8,636 vs. 8,659	79.37% vs. 79.58%	0.023
Hemoglobin	13.073 ± 1.845 vs. 13.331 ± 1.624	13,495 vs. 12,372	81.28% vs. 79.11%	0.149	13.188 ± 1.734 vs. 13.268 ± 1.681	8,603 vs. 8,625	79.06% vs. 79.27%	0.047
Alkaline phosphatase	84.103 ± 43.583 vs. 75.167 ± 33.123	13,170 vs. 12,343	79.33% vs. 78.93%	0.231	79.204 ± 34.991 vs. 77.935 ± 33.539	8,478 vs. 8,494	77.92% vs. 78.06%	0.037
0–50 U/L		1,560 vs. 2,052	9.40% vs. 13.12%	0.118		1,189 vs. 1,199	10.93% vs. 11.02%	0.003
50–70 U/L		4,930 vs. 5,525	29.70% vs. 35.33%	0.121		3,524 vs. 3,520	32.39% vs. 32.35%	0.001
70–90 U/L		5,326 vs. 4,616	32.08% vs. 29.52%	0.056		3,306 vs. 3,349	30.38% vs. 30.78%	0.009
90–120 U/L		3,947 vs. 2,712	23.77% vs. 17.34%	0.160		2,146 vs. 2,144	19.72% vs. 19.70%	0.000
Albumin	3.989 ± 0.489 vs. 4.091 ± 0.422	13,161 vs. 12,397	79.27% vs. 79.28%	0.223	4.052 ± 0.439 vs. 4.064 ± 0.445	8,469 vs. 8,534	77.83% vs. 78.43%	0.026
0–3 g/dL		1,217 vs. 605	7.33% vs. 3.87%	0.151		497 vs. 523	4.57% vs. 4.81%	0.011
3–4 g/dL		7,372 vs. 6,103	44.40% vs. 39.03%	0.109		4,375 vs. 4,382	40.21% vs. 40.27%	0.001
4–5 g/dL		8,985 vs. 9,486	54.12% vs. 60.66%	0.133		6,257 vs. 6,285	57.50% vs. 57.76%	0.005
Bilirubin. total	0.614 ± 0.542 vs. 0.624 ± 0.386	13,108 vs. 12,289	78.95% vs. 78.58%	0.020	0.624 ± 0.510 vs. 0.613 ± 0.374	8,429 vs. 8,459	77.47% vs. 77.74%	0.025
Protein	7.120 ± 0.679 vs. 7.026 ± 0.585	12,927 vs. 12,083	77.86% vs. 77.27%	0.148	7.077 ± 0.627 vs. 7.056 ± 0.592	8,287 vs. 8,325	76.16% vs. 76.51%	0.035
Protein		12,927 vs. 12,083	77.86% vs. 77.27%	0.014		8,287 vs. 8,325	76.16% vs. 76.51%	0.008
Cholesterol, total	183.42 ± 50.17 vs. 180.10 ± 47.12	11,969 vs. 11,427	72.09% vs. 73.07%	0.068	182.66 ± 49.55 vs. 180.52 ± 48.63	7,822 vs. 7,755	71.89% vs. 71.27%	0.044
0–150 mg/dL		2,958 vs. 2,913	17.82% vs. 18.63%	0.021		1,963 vs. 1,984	18.04% vs. 18.23%	0.005
150–200 mg/dL		5,858 vs. 5,804	35.28% vs. 37.12%	0.038		3,824 vs. 3,818	35.14% vs. 35.09%	0.001
200–300 mg/dL		4,672 vs. 4,163	28.14% vs. 26.62%	0.034		2,962 vs. 2,943	27.22% vs. 27.05%	0.004
Cholesterol in HDL	50.799 ± 18.094 vs. 56.082 ± 20.236	11,952 vs. 11,417	71.99% vs. 73.01%	0.275	53.121 ± 18.773 vs. 53.574 ± 19.471	7,821 vs. 7,747	71.88% vs. 71.20%	0.024
0–40 mg/dL		3,366 vs. 2,243	20.27% vs. 14.34%	0.157		1,781 vs. 1,812	16.37% vs. 16.65%	0.008
40–60 mg/dL		6,364 vs. 5,601	38.33% vs. 35.82%	0.052		4,075 vs. 4,059	37.45% vs. 37.30%	0.003
60–80 mg/dL		2,731 vs. 3,458	16.45% vs. 22.11%	0.144		2,059 vs. 2,063	18.92% vs. 18.96%	0.001
Cholesterol in LDL	105.65 ± 39.98 vs. 100.39 ± 36.64	11,883 vs. 11,392	71.58% vs. 72.85%	0.137	104.19 ± 39.19 vs. 102.13 ± 38.17	7,816 vs. 7,718	71.83% vs. 70.93%	0.053
0–50 mg/dL		867 vs. 783	5.22% vs. 5.01%	0.010		560 vs. 571	5.15% vs. 5.25%	0.005
50–100 mg/dL		5,206 vs. 5,710	31.36% vs. 36.51%	0.109		3,591 vs. 3,584	33.00% vs. 32.94%	0.001
100–150 mg/dL		5,672 vs. 5,257	34.16% vs. 33.62%	0.012		3,651 vs. 3,627	33.55% vs. 33.33%	0.005
Triglyceride	141.39 ± 115.20 vs. 119.09 ± 85.01	11,810 vs. 11,368	71.14% vs. 72.69%	0.220	130.17 ± 92.86 vs. 126.22 ± 94.84	7,767 vs. 7,694	71.38% vs. 70.71%	0.042
0–100 mg/dL		5,032 vs. 5,947	30.31% vs. 38.03%	0.163		3,652 vs. 3,649	33.56% vs. 33.54%	0.001
100–150 mg/dL		4,045 vs. 3,883	24.36% vs. 24.83%	0.011		2,646 vs. 2,658	24.32% vs. 24.43%	0.003
150–200 mg/dL		2,334 vs. 1,948	14.06% vs. 12.46%	0.047		1,465 vs. 1,417	13.46% vs. 13.02%	0.013
200–300 mg/dL		1,800 vs. 1,280	10.84% vs. 8.19%	0.091		1,008 vs. 1,008	9.26% vs. 9.26%	0.000
Leukocytes	9.037 ± 93.797 vs. 11.567 ± 147.460	11,360 vs. 10,072	68.42% vs. 64.41%	0.020	9.457 ± 103.863 vs. 10.766 ± 136.414	7,043 vs. 7,112	64.73% vs. 65.36%	0.011
Hemoglobin A1c	6.882 ± 2.024 vs. 6.281 ± 1.452	9,313 vs. 7,508	56.10% vs. 48.01%	0.341	6.513 ± 1.644 vs. 6.413 ± 1.583	5,548 vs. 5,436	50.99% vs. 49.96%	0.062
0–5 %		525 vs. 458	3.16% vs. 2.93%	0.014		320 vs. 338	2.94% vs. 3.11%	0.010
5–6 %		3,924 vs. 3,990	23.64% vs. 25.52%	0.044		2,657 vs. 2,640	24.42% vs. 24.26%	0.004
6–7 %		3,371 vs. 2,927	20.30% vs. 18.72%	0.040		2,155 vs. 2,167	19.80% vs. 19.91%	0.003
7–8 %		1,947 vs. 1,410	11.73% vs. 9.02%	0.089		1,150 vs. 1,140	10.57% vs. 10.48%	0.003
8–9 %		1,249 vs. 733	7.52% vs. 4.69%	0.119		663 vs. 632	6.09% vs. 5.81%	0.012
At least 9 %		1,732 vs. 563	10.43% vs. 3.60%	0.270		548 vs. 549	5.04% vs. 5.04%	0.000
Phosphate	3.664 ± 0.951 vs. 3.543 ± 0.764	3,772 vs. 3,086	22.72% vs. 19.73%	0.141	3.555 ± 0.819 vs. 3.544 ± 0.795	2,196 vs. 2,265	20.18% vs. 20.82%	0.014
Magnesium	1.945 ± 0.334 vs. 1.967 ± 0.304	3,097 vs. 2,555	18.65% vs. 16.34%	0.070	1.956 ± 0.319 vs. 1.952 ± 0.311	1,798 vs. 1,838	16.52% vs. 16.89%	0.014
Ferritin	241.49 ± 526.31 vs. 200.83 ± 561.48	3,103 vs. 2,299	18.69% vs. 14.70%	0.075	200.65 ± 396.23 vs. 201.35 ± 490.87	1,734 vs. 1,733	15.94% vs. 15.93%	0.002
Iron	70.930 ± 41.590 vs. 78.260 ± 37.135	2,953 vs. 2,279	17.79% vs. 14.57%	0.186	75.219 ± 39.526 vs. 76.038 ± 37.949	1,682 vs. 1,677	15.46% vs. 15.41%	0.021
0–50 ug/dL		1,138 vs. 614	6.86% vs. 3.93%	0.130		532 vs. 510	4.89% vs. 4.69%	0.009
50–100 ug/dL		1,691 vs. 1,386	10.19% vs. 8.86%	0.045		995 vs. 1,001	9.14% vs. 9.20%	0.002
100–200 ug/dL		580 vs. 600	3.49% vs. 3.84%	0.018		401 vs. 406	3.69% vs. 3.73%	0.002
C reactive protein	18.166 ± 37.652 vs. 11.456 ± 28.640	2,020 vs. 1,918	12.17% vs. 12.27%	0.201	14.106 ± 32.965 vs. 13.350 ± 30.687	1,248 vs. 1,269	11.47% vs. 11.66%	0.024
0–10 mg/L		1,402 vs. 1,565	8.45% vs. 10.01%	0.054		966 vs. 984	8.88% vs. 9.04%	0.006
10–20 mg/L		455 vs. 293	2.74% vs. 1.87%	0.058		236 vs. 235	2.17% vs. 2.16%	0.001
20–40 mg/L		295 vs. 186	1.78% vs. 1.19%	0.049		149 vs. 147	1.37% vs. 1.35%	0.002

PSM, Propensity Score Matching; VDD, Vitamin D Deficiency; VDA, Vitamin D Adequacy; SD, Standard Deviation; Std. Diff., Standardized Mean Difference; Calcidiol, 25-hydroxyvitamin D; HDL, High-Density Lipoprotein; LDL, Low-Density Lipoprotein; A1c, Hemoglobin A1c; CRP, C-reactive protein.

### 3.2 Kaplan–Meier survival analysis

**Figure 2:**
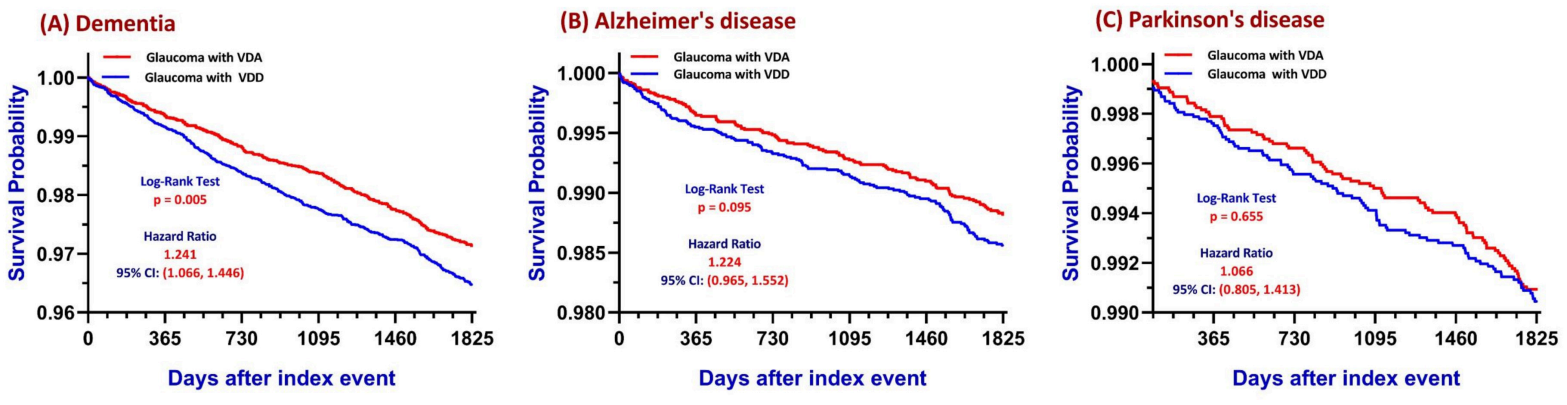
Kaplan–Meier survival curves: neurodegenerative outcomes by vitamin D status in glaucoma patients. Kaplan–Meier survival curves illustrating the cumulative incidence of developing three distinct neurocognitive outcomes in glaucoma patients. The analysis compares patients categorized by their 25-hydroxyvitamin D (25(OH)D) status in a Propensity Score-Matched (PSM) cohort: those with Vitamin D Deficiency (VDD, 25(OH)D < 30 ng/mL) and those with Vitamin D Adequacy (VDA, 25(OH)D ≥30 ng/mL). **(A)** depicts the incidence of Dementia, where the VDD cohort showed a significantly higher cumulative incidence compared to the VDA cohort, with statistical significance determined using an adjusted Cox proportional-hazards model (*p* = 0.005, derived from the log-rank test). **(B, C)** show the incidence of Alzheimer's Disease (AD) and Parkinson's Disease (PD), respectively. For these specific neurodegenerative subtypes, no significant difference in cumulative incidence was observed between the VDD and VDA groups; this non-significant finding is consistent with limitations in statistical power due to lower event rates for AD and PD.

Kaplan–Meier survival analyses demonstrated a significant difference in the primary outcome, unspecified dementia, between glaucoma patients with VDD and those with VDA over the 5-year follow-up period, as shown in Figure 2A.

VDD was associated with a higher risk of unspecified dementia, with a significantly lower survival probability throughout the observation window (Log-Rank chi-squared = 7.742; *p* = 0.005) and an increased hazard of disease (HR = 1.241; 95% CI: 1.066–1.446) (Figure 2A). The absolute 5-year risk was 3.2% in the VDD cohort vs. 2.6% in the VDA cohort (Supplementary Table S1).

By contrast, there was no statistically significant difference in the incidence of AD (Figure 2B) or PD (Figure 2C). For AD, the association was non-significant (HR 1.224; 95% CI: 0.965–1.552; *p* = 0.095). Similarly, PD incidence was nearly identical between the two groups (HR 1.066; 95% CI: 0.805–1.413; *p* = 0.655).

### 3.3 Landmark analyses

Landmark Kaplan–Meier analyses comparing glaucoma patients with VDD and VDA were conducted at 1, 3, and 5 years following the index diagnosis (Table 2).

**Table 2:** Landmark analysis: time-dependent hazard ratios for neurodegenerative outcomes.

**Follow up**	**Outcomes**	**Cohorts**	**Patients in cohort**	**Patients with outcome**	**Survival probability**	**Hazard ratio**	**95% CI**	**Log-rank test *P*-value**
1 year	Dementia	VDD	10,463	88	99.137%	1.323	(0.963–1.819)	0.083
		VDA	10,465	67	99.348%			
3 years		VDD	10,466	225	97.702%	1.396	(1.142–1.707)	0.001
		VDA	10,466	164	98.352%			
5 years		VDD	11,478	365	96.48%	1.241	(1.066–1.446)	0.005
		VDA	11,479	301	97.14%			
1 year	Alzheimer's disease	VDD	10,559	47	99.544%	1.314	(0.852–2.029)	0.216
		VDA	10,547	36	99.652%			
3 years		VDD	10,559	89	99.103%	1.157	(0.854–1.569)	0.345
		VDA	10,549	78	99.222%			
5 years		VDD	11,573	150	98.56%	1.224	(0.965–1.552)	0.095
		VDA	11,570	125	98.82%			
1 year	Parkinson's disease	VDD	10,518	25	99.757%	1.401	(0.765–2.569)	0.273
		VDA	10,542	18	99.826%			
3 years		VDD	10,519	57	99.42%	1.234	(0.839–1.816)	0.285
		VDA	10,543	47	99.529%			
5 years		VDD	11,532	99	99.04%	1.066	(0.805–1.413)	0.655
		VDA	11,568	95	99.09%			

This table presents the results of the landmark sensitivity analysis, detailing the Hazard Ratios (HRs) and 95% Confidence Intervals (CIs) for the association between Vitamin D Deficiency (VDD, < 30 ng/mL) and incident neurodegenerative outcomes, compared to the Vitamin D Adequacy (VDA, ≥30 ng/mL) group. The risk is assessed at specific follow-up time points: 1, 3, and 5 years.

This methodology was employed to evaluate the stability and persistence of the dementia, Alzheimer's disease, and Parkinson's disease risks associated with VDD across the observation period. The analysis helps determine if the risk persists beyond the initial diagnosis or if it is confined to the early stages following index date.

HR, Hazard Ratio; CI, Confidence Interval; VDD, Vitamin D Deficiency; VDA, Vitamin D Adequacy.

The excess risk of dementia associated with VDD strengthened over the follow-up period. At 1 year, the difference was not yet statistically significant (HR 1.323, 95% CI 0.963–1.819; *p* = 0.083). However, the elevated risk became significant at the 3-year landmark (HR 1.396; *p* = 0.001) and remained significant at 5 years (HR 1.241; 95% CI 1.066–1.446; *p* = 0.005).

Across all landmark analyses, the incidence of AD and PD did not show statistically significant survival differences, indicating that the impact of VDD is primarily associated with the broader dementia diagnosis and the effect is most pronounced in the mid-to-long term.

### 3.4 Subgroup analyses

**Figure 3:**
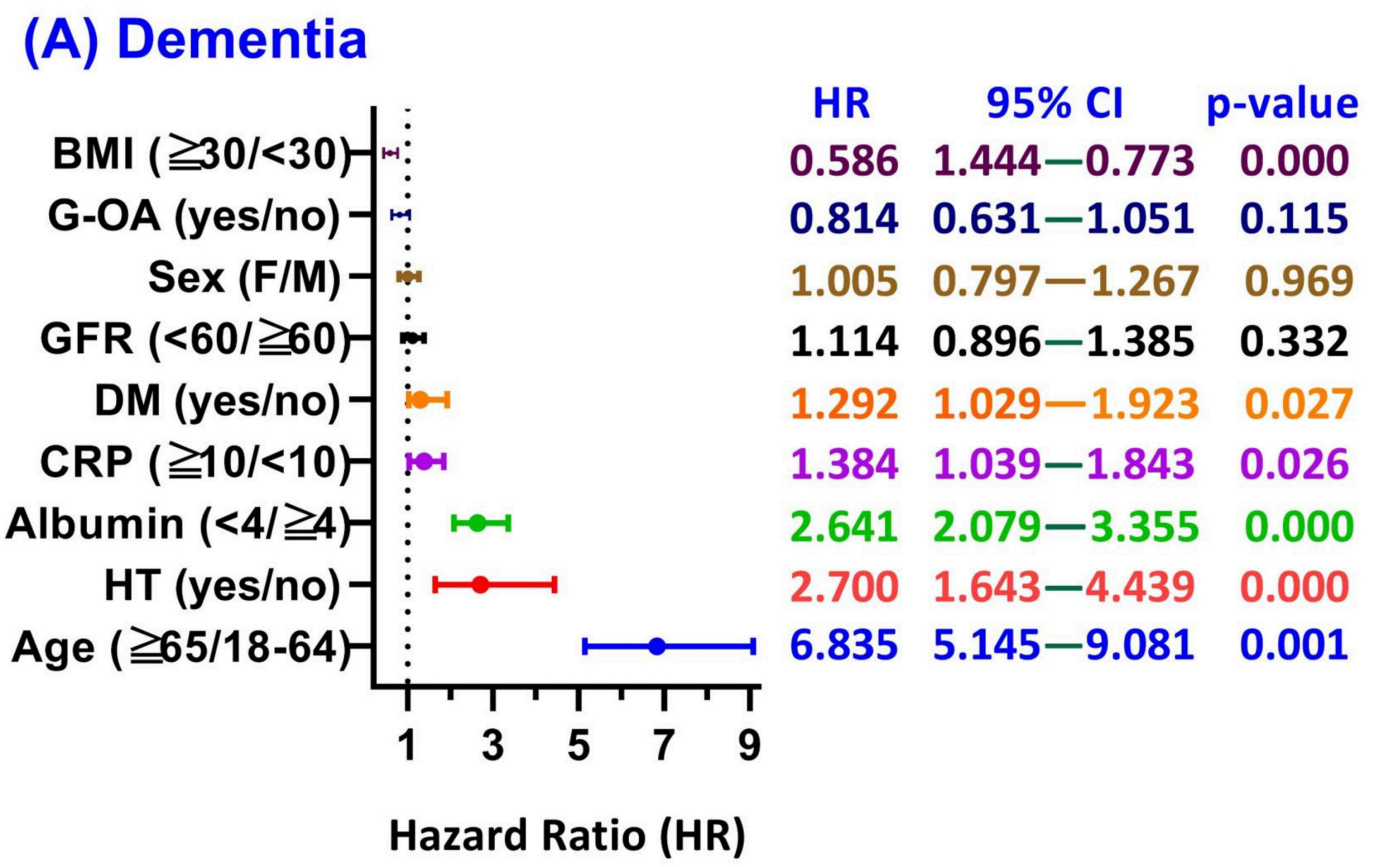
Subgroup Analysis of 5-Year Dementia Risk Stratified by Vitamin D Status in Glaucoma Patients. Forest plot presenting hazard ratios (HR) for the 5-year risk of incident dementia, comparing the Vitamin D Deficiency (VDD, < 30 ng/mL) cohort against the Vitamin D Adequacy (VDA, ≥30 ng/mL) cohort across various clinical subgroups. The VDA group serves as the reference for all HR calculations. Each row displays the HR and its 95% Confidence Interval (CI). The vertical dashed line at HR = 1.0 indicates the null value. All HRs were calculated using Cox proportional-hazards models adjusted for age, sex, and race/ethnicity, highlighting the consistent association across demographics, cardiometabolic conditions (e.g., diabetes, hypertension), and inflammatory markers (e.g., CRP). G-OA, Open-angle glaucoma; VDD, Vitamin D Deficiency; VDA, Vitamin D Adequacy; HR, Hazard Ratio; CI, Confidence Interval; CRP, C-Reactive Protein; GFR, Glomerular Filtration Rate; BMI, Body Mass Index; DM, Diabetes Mellitus; HT, Hypertension.

Due to limited case counts for specific outcomes, the subgroup analysis (Figure 3) and effect modification assessment were performed exclusively for unspecified dementia. Analysis across patient subgroups revealed that the 5-year dementia risk associated with VDD was markedly influenced by the presence of specific baseline characteristics.

Advanced age (65 years or older vs. 18–64 years) emerged as the most significant predictor (HR 6.84, 95% CI 5.15–9.08; *p* = 0.0001). Other major risk factors included hypertension (HR 2.70, 1.64–4.44; *p* < 0.0001), hypoalbuminemia (albumin < 4 vs. ≥4 g/dL: HR 2.64, 2.08–3.36; *p* < 0.0001), elevated inflammatory burden (CRP level 10 mg/L or greater vs. less than 10 mg/L; HR 1.38, 1.04–1.84; *p* = 0.026), and diabetes (HR 1.29, 1.03–1.92; *p* = 0.027). Conversely, higher BMI (30 kg/m^2^ or greater vs. less than 30 kg/m^2^) was associated with a paradoxical lower observed risk (HR 0.59, 0.44–0.77; *p* < 0.001). Glaucoma subtype was not a significantly associated with dementia risk (HR = 0.814; 95% CI: 0.63–1.05; *p* = 0.115).

### 3.5 Sensitivity and detection bias assessment

Sensitivity analyses confirmed the robustness of the primary findings under varied conditions. Before propensity score matching (PSM), VDD was associated with a lower hazard of unspecified dementia (HR = 0.871; 95% CI: 0.762–0.995; *P* = 0.042), which reversed after matching, showing a higher hazard (HR = 1.241; 95% CI: 1.066–1.446; *P* = 0.005) (Supplementary Table S2). Applying a stricter deficiency threshold (25[OH]D < 20 ng/mL) revealed a stronger association with dementia risk (HR = 1.493; 95% CI: 1.179–1.890; *P* < 0.001) than the primary threshold of < 30 ng/mL (Supplementary Table S3). Excluding long-term NSAID users attenuated the association, rendering it non-significant (HR = 1.178; 95% CI: 0.998–1.391; *P* = 0.053) (Supplementary Table S4). Finally, healthcare utilization during follow-up was examined (Supplementary Tables S5–S7). After matching, the VDA cohort had a higher baseline visit frequency (120.49 vs. 110.86 visits; *P* < 0.0001; Supplementary Table S5), while 5-year hospitalization rates were similar between groups (1.93 vs. 1.89 visits; *P* = 0.641; Supplementary Table S6). The VDA group also exhibited more ophthalmology-related encounters and procedures than the VDD group (3.17 vs. 2.87 visits; *P* < 0.0001; Supplementary Table S7).

The restricted sensitivity analysis confirmed that the hazard ratio remained stable when matching was limited to high-completeness variables (HR 1.239, *p* = 0.008; Supplementary Table S8).

## 4 Discussion

This large-scale retrospective cohort study, utilizing a PSM cohort of glaucoma patients, establishes a significant and independent association between VDD (25(OH)D < 30 ng/mL) and an increased hazard of developing unspecified dementia (HR 1.241; *P* = 0.005). This result suggests that VDD confers a modest, yet clinically meaningful, long-term increase in dementia risk within this vulnerable neurodegenerative population. This pattern is consistent with existing epidemiological literature, which reports the strongest risk association for VDD with unspecified dementia (25, 28), and inconsistent or null association with PD (29, 30).

The necessity of the PSM procedure is validated by the elimination of a substantial bias. Prior to PSM, VDD falsely suggested a protective effect (HR 0.871; *p* = 0.042); the correction revealed a significant, harmful association (HR 1.241; *p* = 0.005). Furthermore, the dose-response relationship confirmed in the sensitivity analysis, where a more severe deficiency (25(OH)D < 20 ng/mL) yielded a notably stronger signal (HR 1.493; *p* < 0.001), supports validity of this core finding.

### 4.1 Biological plausibility and mechanisms

The association between VDD and increased dementia risk is highly consistent with the known pleiotropic, non-skeletal actions of vitamin D. 1,25-dihydroxyvitamin D functions as a crucial steroid hormone by binding the VDR, which is widely expressed throughout the CNS, including the hippocampus, cortex, and key ocular neuroglial elements like retinal ganglion cells and optic nerve head astrocytes (19, 28, 31).

Vitamin D supports neural protection by aiding amyloid clearance and reducing neuronal toxicity, while deficiency may promote vascular dysfunction and brain atrophy associated with cognitive decline (27, 32) In neurological tissues, active vitamin D signaling regulates processes critical to neurodegeneration by down-modulating chronic inflammation and oxidative stress, such as suppressing the NF-kappa-B signaling pathway (18, 33). Since both glaucoma and dementia involve chronic low-grade neuroinflammation (34–37), VDD may act as an inflammatory sensitizer, compounding neuronal vulnerability and accelerating CNS decline (18, 38).

### 4.2 Systemic vulnerability and modifying factors

The inflammatory mechanism proposed is strongly supported by the sensitivity analysis focusing on NSAID use. Long-term NSAID use is epidemiologically linked to reduced dementia risk, and the protective effect appears more pronounced for NSAIDs without amyloid-β lowering properties, and cumulative dose does not play a major role (39, 40). The finding that the association between vitamin D deficiency and dementia risk diminishes to non-significance after excluding long-term NSAID users underscores a potentially complex relationship. It suggests that the anti-inflammatory effects of NSAIDs might not effectively reduce or could even influence the excess dementia risk linked to vitamin D deficiency, possibly acting as a systemic inflammation proxy or marker (41, 42). This attenuation indicates that long-term NSAID use may confound or modify the relationship between vitamin D status and dementia, emphasizing the role of systemic inflammation in dementia pathogenesis. Limited data exist on how vitamin D deficiency and NSAID use interact in relation to dementia risk, with no strong evidence for a synergistic or antagonistic effect. Current evidence suggests vitamin D deficiency raises the risk of dementia, while long-term NSAID use may modestly reduce it, likely via anti-inflammatory mechanisms (41, 42). However, NSAIDs are not recommended clinically for dementia prevention due to their potential adverse effects and inconsistent findings across studies, with research often limited to prescription use and possibly missing over-the-counter consumption (39).

Subgroup analysis revealed that the dementia risk associated with VDD is highly dependent on underlying systemic health. Markers of advanced age (HR 6.84), hypertension (HR 2.70), and hypoalbuminemia (HR 2.64) were powerful predictors, suggesting that VDD may function primarily as an indicator of an overall vulnerable systemic profile rather than the sole driver of pathology (28, 43).

Also, the elevated risk was also pronounced in patients with markers of systemic inflammation: elevated CRP (≥10 mg/L) (HR 1.38) and diabetes (HR 1.29), reinforcing that VDD compounds risk in individuals with high inflammatory and metabolic burdens, pointing toward a shared pathway involving microvascular compromise (44–46). The finding that higher BMI (≥30 kg/m^2^) was associated with a paradoxical lower observed risk (HR 0.59) must be interpreted with caution. Potential explanations include reverse causation, where pre-clinical dementia leads to weight loss, or that higher BMI may reflect better underlying nutritional reserves acting as a buffer against severe systemic decline and frailty (47, 48).

On the contrary, our analysis showed that glaucoma subtype (PACG vs. POAG) did not significantly influence the hazard for dementia (HR = 0.814, *p* = 0.115). This contrast emphasizes that systemic metabolic and inflammatory factors, rather than local ophthalmic classifications, are the primary drivers of neurodegenerative outcomes in this population.

### 4.3 Subtype findings and residual confounding

The decision to focus on “unspecified dementia” is validated by clinical practice: many patients initially present with cognitive decline that cannot be confidently classified, and thus “unspecified” or mixed dementia diagnoses account for the majority of incident cases (49, 50). These early, heterogeneous presentations provide greater statistical power. Conversely, the lower event rates for specific neurodegenerative subtypes (AD and PD) in our cohort resulted in limited power to detect subtype-specific associations.

Although comprehensive PSM accounted for multiple covariates, differences in healthcare utilization persisted. The VDA cohort showed higher frequencies of hospital visits and ophthalmic services (Supplementary Tables S5 and S7), which may reflect an underlying tendency for greater healthcare engagement or a different comorbidity profile—factors often cited as sources of unmeasured confounding in large-scale EHR studies (51–53). However, rather than weakening the results, this pattern strengthens their validity. Greater clinical contact in the VDA group would typically increase the chance of dementia detection, yet the VDD cohort still exhibited a higher dementia risk. This suggests that detection bias is unlikely to explain the association and, if present, would have reduced rather than exaggerated the observed effect. Comparable hospitalization rates between cohorts (Supplementary Table S6) further indicate that the findings are not driven by greater systemic disease burden in the VDD group. Consistent results across sensitivity analyses reinforce the robustness of these findings. When limiting the propensity score model to 26 high-completeness variables (Supplementary Table S8), the estimated hazard ratio for dementia remained virtually unchanged, confirming that the association between vitamin D deficiency and dementia risk is stable and not materially influenced by data completeness or model specification.

### 4.4 Limitations

This retrospective EHR study has inherent limitations precluding causal inference, including reliance on ICD-10 codes prone to inaccuracies, a single baseline 25(OH)D measure omitting longitudinal changes, and absence of granular ophthalmic staging (e.g., visual field indices, cup-to-disc ratios) despite no glaucoma subtype-dementia interaction (*P* = 0.115). The “unspecified dementia” endpoint limits etiological specificity; however, supplementary analyses refute detection bias from differential healthcare use, as the VDA group had more ophthalmology encounters (*P* < 0.0001). Although 1:1 propensity score matching and no multiple imputation were used, excellent covariate balance (all SMDs < 0.1), large sample size, and stable sensitivity analyses support robust, clinically relevant findings despite potential generalizability constraints from the federated EHR platform.

## 5 Conclusion

This large-scale, PSM-matched retrospective cohort study suggests that VDD is associated with an increased likelihood for developing unspecified dementia in patients with primary glaucoma (HR 1.241). This elevated risk, which is amplified by concurrent systemic inflammation and cardiometabolic diseases but not reduced by long-term NSAIDs, highlights the importance of considering a systemic approach in glaucoma management. Our findings support integrating vitamin D assessment and optimization into clinical care to potentially reduce neurocognitive decline. Given the observational nature of this cohort, further prospective research is warranted to account for potential residual confounding and ascertainment bias, and to clarify the therapeutic efficacy of vitamin D supplementation.

## Data Availability

Due to licensing and privacy restrictions, the de-identified, aggregate-level data used in this study from the TriNetX Global Health Research Network are not publicly available. TriNetX provides access to data sourced from a global network of healthcare organizations. Researchers may request access through the TriNetX website (https://trinetx.com) or by contacting Privacy@TriNetX.com. Data are also available from the corresponding author upon reasonable request. Requests to access the dataset can be made directly to TriNetX through their official website: https://www.trinetx.com/contact/.
