# Supplementary material for: Increased risk of incident dementia associated with vitamin D deficiency in glaucoma patients: a TriNetX cohort study
Source: Front Nutr. 2026 Feb 10;13:1760959. doi: 10.3389/fnut.2026.1760959 (PMC12929110; doi:10.3389/fnut.2026.1760959)
Supplement: Supplementary file 1 [file Table_1.docx]

**Supplementary Table S1. Five-Year Risks and Relative Associations for Neurodegenerative Outcomes Stratified by Vitamin D Status in Glaucoma Patients** **(Before Propensity Score Matching).**

The analysis presents crude (unadjusted) Hazard Ratios (HRs) and 95% Confidence Intervals (CIs) comparing the raw cohorts of glaucoma patients with Vitamin D Deficiency (VDD, <30 ng/mL) versus Vitamin D Adequacy (VDA, ≥30 ng/mL) prior to Propensity Score Matching (PSM).

**
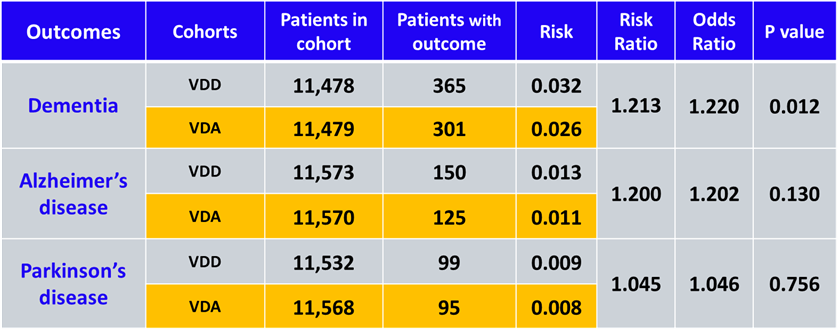
**

*Note. Values are based on the initial cohort before adjusting for covariates. HRs represent the relative risk of the VDD group compared to the VDA reference group.*

**Abbreviations**: HR, Hazard Ratio; CI, Confidence Interval; VDD, Vitamin D Deficiency; VDA, Vitamin D Adequacy; PSM, Propensity Score Matching.

**Supplementary Table S2. Comparison of 5-Year Survival Analysis Results Before and After Propensity Score Matching (PSM)**

This table provides a comparative summary of 5-year risk estimates for incident dementia, Alzheimer’s disease, and Parkinson’s disease. It contrasts results from the unadjusted raw cohorts with those from the 1:1 PSM cohorts to demonstrate the consistency of the association between Vitamin D Deficiency (VDD, <30 ng/mL) and neurodegenerative outcomes after adjusting for baseline confounders.


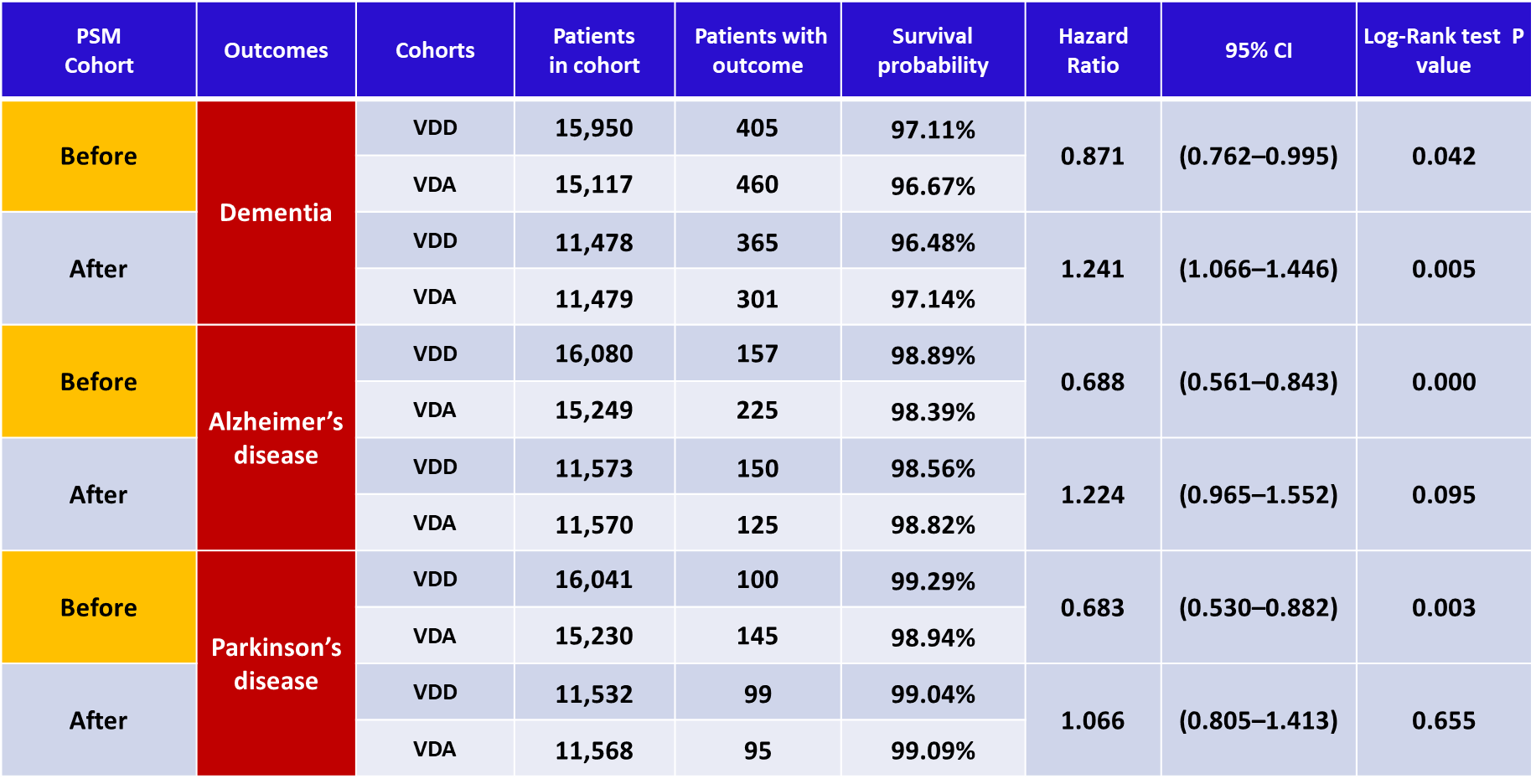


*Note. The VDA group (≥30 ng/mL) serves as the reference category. KM survival probabilities represent the 5-year event-free rates.*

**Abbreviations:** HR, Hazard Ratio; CI, Confidence Interval; VDD, Vitamin D Deficiency; VDA, Vitamin D Adequacy; PSM, Propensity Score Matching; KM, Kaplan–Meier.

**Supplementary Table S3. Sensitivity Analysis: Dose-Response Relationship Between Vitamin D Deficiency Severity and 5-Year Neurodegenerative Risks.**

This table presents hazard ratios (HRs) and 95% confidence intervals (CIs) for neurodegenerative outcomes using different clinical thresholds for Vitamin D status. It compares Severe Vitamin D Deficiency (VDD, <20 ng/mL) and General VDD (<30 ng/mL) against the Vitamin D Adequacy (VDA, ≥30 ng/mL) reference group. These findings confirm that the increased risk for unspecified dementia is sustained and amplified (showing a potential dose-response relationship) when applying a more stringent <20 ng/mL threshold.


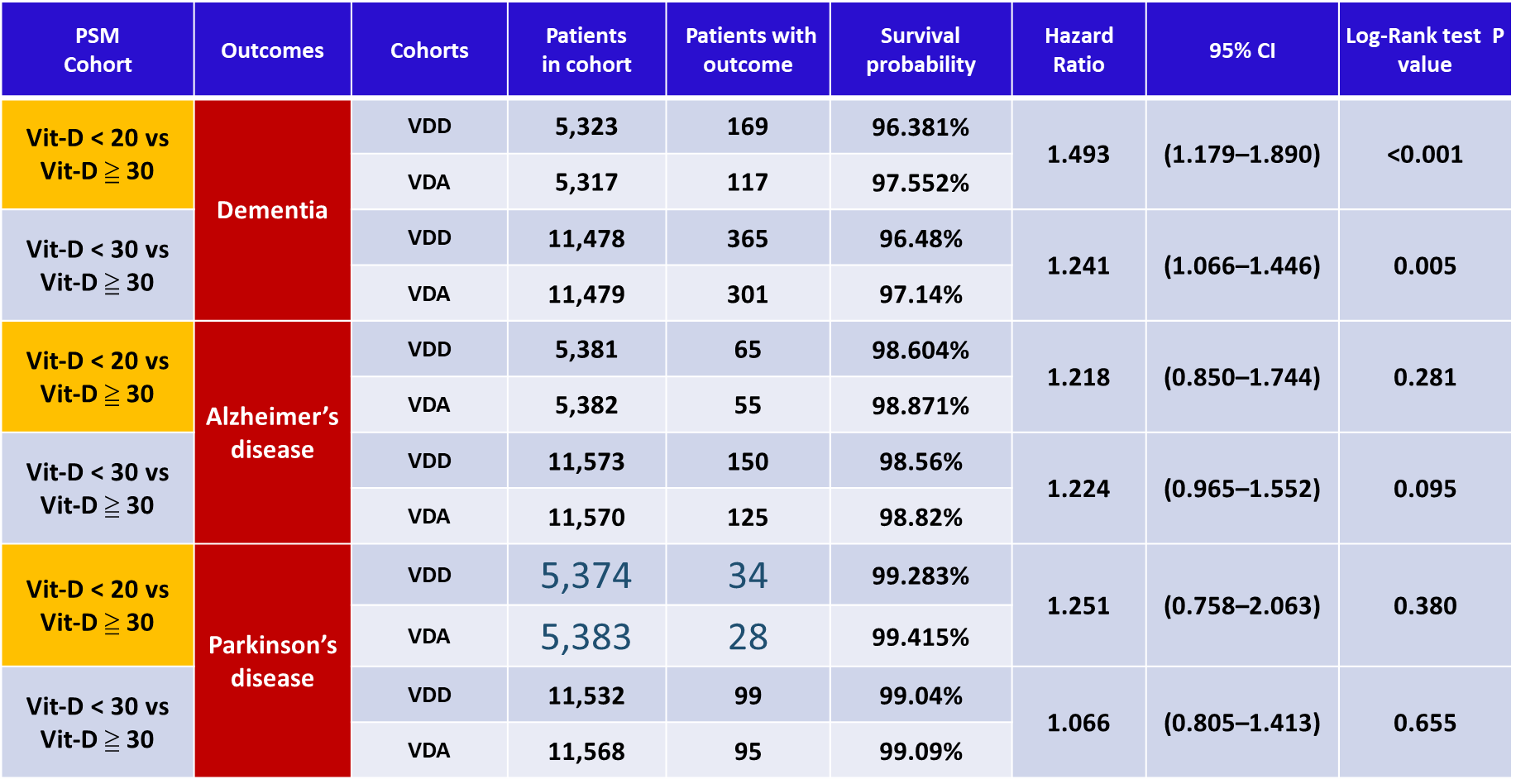


*Note. The VDA group (≥30 ng/mL) serves as the reference category for all hazard ratio calculations*

**Abbreviations:** HR, Hazard Ratio; CI, Confidence Interval; VDD, Vitamin D Deficiency; VDA, Vitamin D Adequacy

**Supplementary Table S4. Sensitivity Analysis: 5-Year Neurodegenerative Risks in Glaucoma Patients, Excluding Long-Term NSAID Users.**

This table presents 5-year risk estimates and survival probabilities for incident dementia, Alzheimer’s disease, and Parkinson’s disease within a restricted cohort that systematically excludes long-term NSAID users. This analysis evaluates whether the observed associations are independent of the potential anti-inflammatory effects of chronic NSAID use. The attenuation of risk estimates in this subgroup suggests that systemic inflammatory pathways—potentially modulated by both vitamin D and NSAIDs—play a role in the link between VDD and dementia risk.


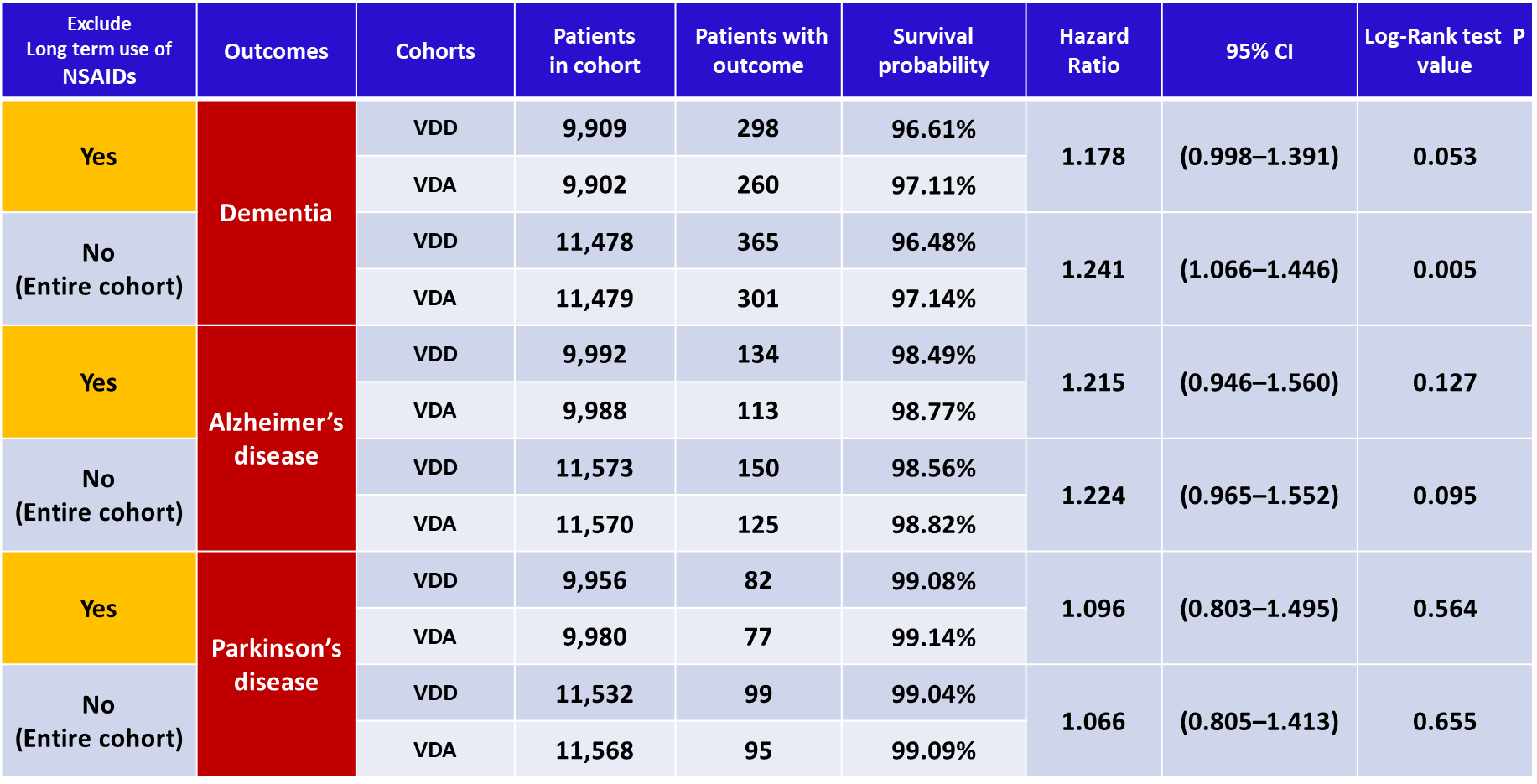


*Note. The VDA group (≥30 ng/mL) serves as the reference category. Long-term NSAID use was defined as per the study's pre-specified criteria in the Methods section.*

**Abbreviations:** HR, Hazard Ratio; CI, Confidence Interval; VDD, Vitamin D Deficiency; VDA, Vitamin D Adequacy; NSAIDs, Non-Steroidal Anti-Inflammatory Drugs.

**Supplementary Table S5. Analysis of Healthcare Utilization: Comparison of Hospital Visits Between VDD and VDA Cohorts.**

This table evaluates potential detection bias by comparing the average number of hospital visits between the Vitamin D Deficiency (VDD, <30 ng/mL) and Vitamin D Adequacy (VDA, ≥30 ng/mL) cohorts. Both raw (unmatched) and 1:1 PSM-balanced data are presented. In both analyses, the VDA cohort demonstrated a significantly higher mean number of visits (p < 0.0001). This consistent finding indicates that the lower dementia risk observed in the VDA group cannot be attributed to lower medical surveillance; rather, the VDA group was more frequently monitored.


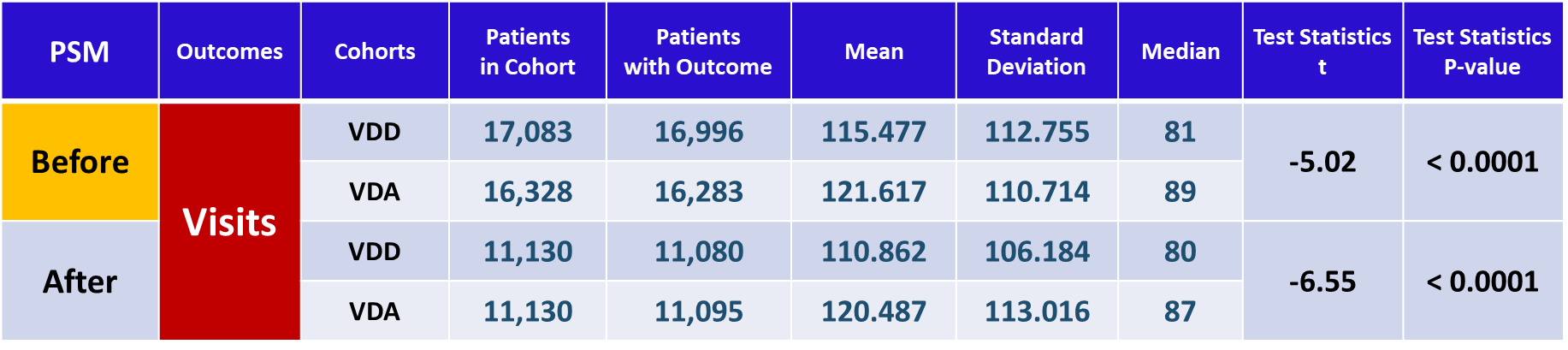


*Note. Statistical significance was determined using the independent t-test. A higher number of visits represents greater healthcare utilization.*

**Abbreviations**: VDD, Vitamin D Deficiency; VDA, Vitamin D Adequacy; PSM, Propensity Score Matching; SD, Standard Deviation.

**Supplementary Table S6. Systemic Healthcare Utilization (Hospitalizations) in Propensity Score-Matched Glaucoma Cohorts by Vitamin D Status.**

This table compares systemic healthcare utilization, measured by the frequency and proportion of hospitalizations over a 5-year follow-up period. It evaluates whether the Vitamin D Deficiency (VDD, <30 ng/mL) and Vitamin D Adequacy (VDA, ≥30 ng/mL) cohorts differ in their overall medical burden. "Patients with outcome" indicates those who experienced at least one hospitalization. The **non-significant P-value (0.641)** demonstrates that both groups had comparable baseline health-seeking behaviors and systemic medical burdens, further mitigating concerns regarding healthcare utilization bias.


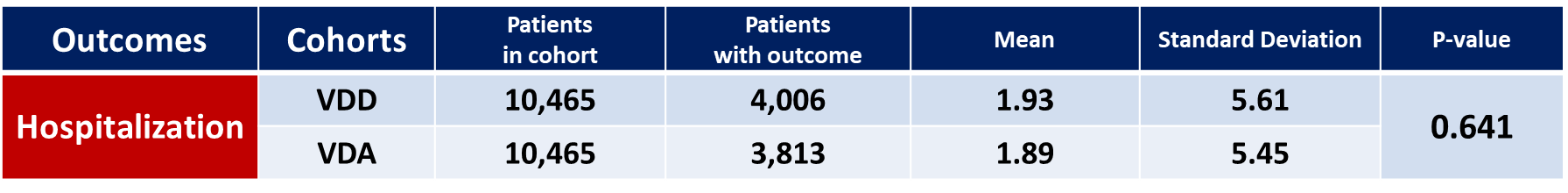


*Note. Statistical significance was assessed using the independent t-test for the frequency of events and the Chi-square test for the proportion of patients hospitalized. A non-significant result indicates well-balanced cohorts regarding acute medical care needs.*

**Abbreviations:** VDD, vitamin D deficiency; VDA, vitamin D adequate

**Supplementary Table S7. Ophthalmology-Related Healthcare Utilization and Procedure Frequency in Propensity Score-Matched Glaucoma Cohorts by Vitamin D Status.**

This table compares the frequency of ophthalmology-specific healthcare services and procedures over a 5-year follow-up period between the Vitamin D Deficiency (VDD) and Vitamin D Adequacy (VDA) cohorts. "Patients with outcome" refers to individuals who underwent at least one ophthalmology-related service or procedure, while the mean and standard deviation represent the average number of encounters per patient. Statistical significance was determined using an independent t-test for frequency and a Chi-square test for proportions. Although the VDD group showed a statistically lower mean utilization rate compared to the VDA group (2.87 vs. 3.17, P < 0.0001), the absolute difference is minimal (0.3 encounters over 5 years). This finding suggests that the higher hazard for dementia observed in the VDD group is not driven by increased clinical surveillance or detection bias.


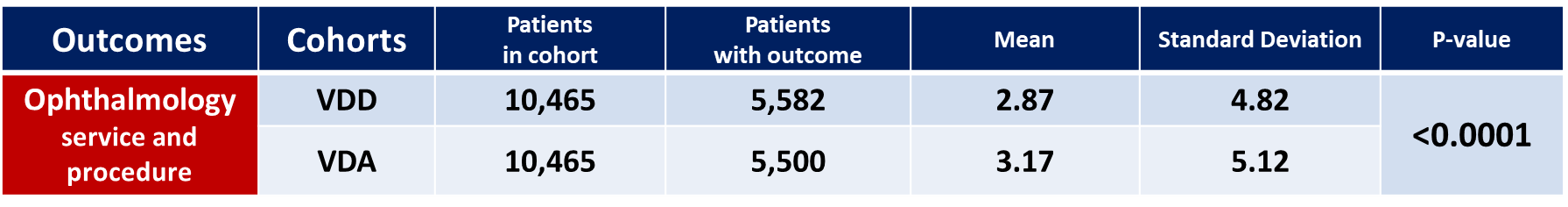


*Note. Statistical significance was determined using the independent t-test for frequency and the Chi-square test for proportions. Despite the small p-value, the absolute difference of 0.3 visits over 5 years is considered clinically negligible regarding detection bias.*

**Abbreviations:** VDD, vitamin D deficiency; VDA, vitamin D adequacy

**Supplementary Table S8. Sensitivity Analysis: Sensitivity Analysis: Stability of Hazard Ratios Across Different Propensity Score Matching Specifications.**

This table presents a comparison of hazard ratios (HR) and 95% confidence intervals (CI) for dementia, Alzheimer’s disease, and Parkinson’s disease between the Vitamin D Deficiency (VDD, <30 ng/mL) and Vitamin D Adequacy (VDA, ≥30 ng/mL) cohorts using two distinct propensity score matching specifications. The Full Model incorporates all 46 identified baseline demographics, comorbidities, and the complete set of 26 laboratory variables, while the Restricted Model is limited to 26 covariates including demographics, diagnoses, medications, and only the 6 laboratory variables with high data completeness (>85% availability). Data are presented as Hazard Ratio (95% Confidence Interval) calculated via Cox proportional hazards regression, with P-values derived from the log-rank test where a value below 0.05 indicates statistical significance.


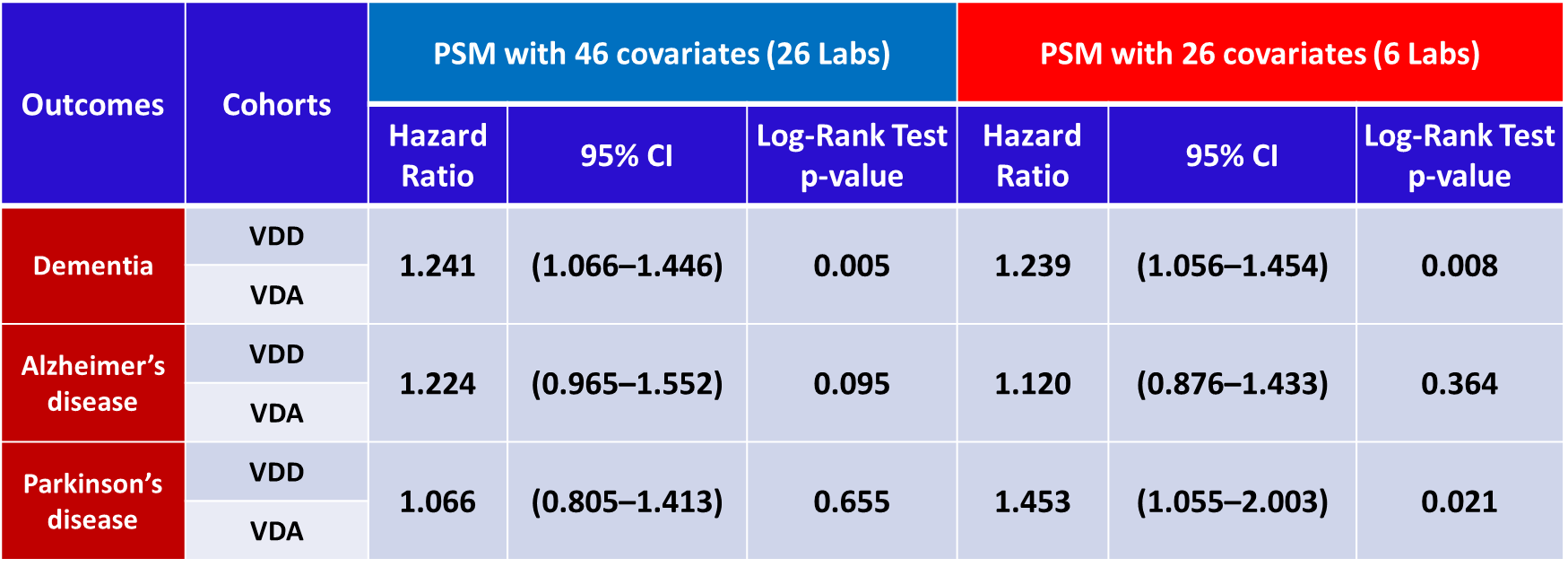


*Note. Data are presented as HR (95% CI). All HRs were calculated via Cox proportional hazards regression. Statistical significance (p < 0.05) was determined using the log-rank test within the matched cohorts.*

**Abbreviations:** VDD, vitamin D deficiency; VDA, vitamin D adequacy; PSM, propensity score matching; HR, hazard ratio; CI, confidence interval; Labs, laboratory variables.

**Supplementary Figure S1. Propensity Score Density Distributions Before and After Matching.**

This figure illustrates the distribution of propensity scores for the Vitamin D Deficiency (VDD; purple line) and Vitamin D Adequacy (VDA; green line) cohorts. Before matching (left panel), distinct propensity score distributions are observed, reflecting significant baseline imbalances between the groups. After 1:1 propensity score matching (right panel), the density curves overlap almost entirely, demonstrating that effective balance across all 46 covariates was achieved and that comparisons are restricted to the region of common support. This high degree of overlap ensures that the observed associations are not driven by baseline demographic or clinical differences.


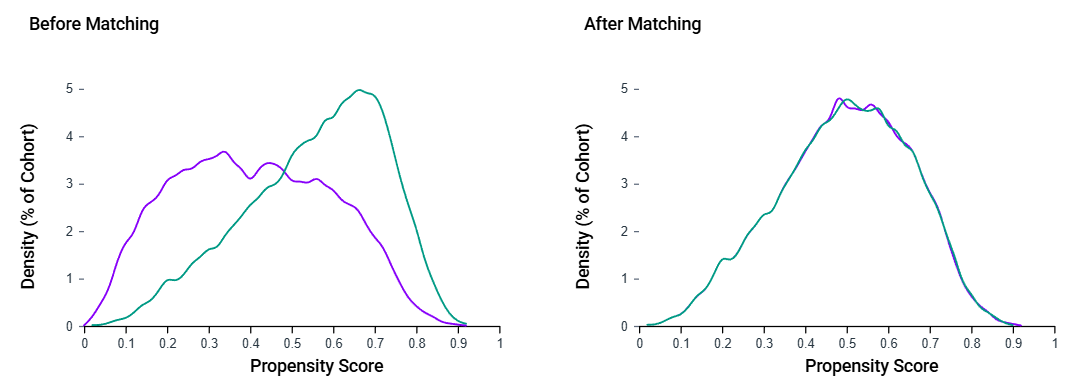


*Note. Data are presented as HR (95% CI). All HRs were calculated via Cox proportional hazards regression. Statistical significance (p < 0.05) was determined using the log-rank test within the matched cohorts.*

**Abbreviations:** **VDD**, vitamin D deficiency (<30 ng/mL); **VDA**, vitamin D adequacy (≥30 ng/mL); **PSM**, propensity score matching; **HR**, hazard ratio; **CI**, confidence interval; **Labs**, laboratory variables.
